# Convolutional Models with Multi-Feature Fusion for Effective Link Prediction in Knowledge Graph Embedding

**DOI:** 10.3390/e25101472

**Published:** 2023-10-21

**Authors:** Qinglang Guo, Yong Liao, Zhe Li, Hui Lin, Shenglin Liang

**Affiliations:** 1 School of Cyber Science and Technology, University of Science and Technology of China, Heifei 230027, China; 2 National Engineering Research Center for Public Safety Risk Perception and Control by Big Data (RPP), CETC Academy of Electronics and Information Technology Group Co., Ltd., China Academic of Electronics and Information Technology, Beijing 100041, China; linhui@cetc.com.cn; 3 Department of Electrical and Electronic Engineering, The Hong Kong Polytechnic University, Hong Kong SAR, China; 4 School of Telecommunications Engineering, Xidian University, Xi’an 710071, China

**Keywords:** link prediction, knowledge graph embeddings, convolution-based

## Abstract

Link prediction remains paramount in knowledge graph embedding (KGE), aiming to discern obscured or non-manifest relationships within a given knowledge graph (KG). Despite the critical nature of this endeavor, contemporary methodologies grapple with notable constraints, predominantly in terms of computational overhead and the intricacy of encapsulating multifaceted relationships. This paper introduces a sophisticated approach that amalgamates convolutional operators with pertinent graph structural information. By meticulously integrating information pertinent to entities and their immediate relational neighbors, we enhance the performance of the convolutional model, culminating in an averaged embedding ensuing from the convolution across entities and their proximal nodes. Significantly, our methodology presents a distinctive avenue, facilitating the inclusion of edge-specific data into the convolutional model’s input, thus endowing users with the latitude to calibrate the model’s architecture and parameters congruent with their specific dataset. Empirical evaluations underscore the ascendancy of our proposition over extant convolution-based link prediction benchmarks, particularly evident across the FB15k, WN18, and YAGO3-10 datasets. The primary objective of this research lies in forging KGE link prediction methodologies imbued with heightened efficiency and adeptness, thereby addressing salient challenges inherent to real-world applications.

## 1. Introduction

Link prediction is a critical task in the field of knowledge graph embedding (KGE) to uncover potential missing or unknown relationships between entities in a knowledge graph (KG) [1,2,3]. Knowledge graphs are structured representations of real-world knowledge, where entities are nodes and their relationships are edges. The primary objective of link prediction is to infer new edges by leveraging the existing graph structure, entity attributes, or embeddings obtained with KGE methods. The importance of link prediction stems from its broad range of applications across various domains, such as social network analysis [4,5,6], recommender systems [7,8], bioinformatics [9], and knowledge base completion (KBC) [10,11]. By identifying missing or unknown links, link prediction enhances the quality and completeness of knowledge graphs, enabling more effective reasoning and decision making in various AI and NLP tasks [12,13,14,15].

Link prediction in knowledge graph embedding presents several challenges that must be addressed to ensure their effectiveness in real-world scenarios. One critical aspect of these challenges is ensuring that link predictors scale in a manageable way regarding the number of parameters and computational costs. Real-world knowledge graphs often consist of millions of entities and relations, resulting in considerable computational overhead for link predictors [2]. Designing link prediction algorithms that can efficiently handle large-scale knowledge graphs is crucial for their practical applicability. Many existing link prediction methods involve complex models with numerous parameters, especially graph-based neural networks, such as GCNs [16] and R-GCNs [17]. Managing the number of parameters is essential to avoid overfitting and ensure the efficient training of models on large-scale knowledge graphs. The link prediction process can be computationally expensive due to the need to compute scores for many potential links. Developing link prediction methods with reduced computational costs is essential for their deployment in real-world applications, especially when working with large-scale knowledge graphs and limited computational resources.

Previous research has attempted to address the challenges associated with link prediction scalability in knowledge graph embeddings. However, despite these efforts, some limitations still exist. Techniques like negative sampling have been adopted to reduce the computational cost by limiting the number of negative samples used during training [18,19]. While negative sampling can significantly decrease computational costs, it may introduce sampling bias and result in sub-optimal model performance. To address the computational cost and large-scale knowledge graph challenges, approximate methods such as locality-sensitive hashing (LSH) [20] and adaptive sampling [21] have been employed. While these methods can reduce the computational cost, they may introduce approximation errors and compromise the quality of predictions. Some research has focused on developing simplified models, such as DistMult [22] and ComplEx [23], which maintain fewer parameters and require less computation. However, the trade-off between model complexity and expressiveness can lead to limitations in capturing more complex relationships and patterns in the data. Incremental learning techniques, such as Know-Evolve [24] and EvolveGCN [25], have been proposed to address the challenge of dynamic updates in knowledge graphs. These methods update the embeddings online without retraining the entire model. While incremental learning can efficiently handle dynamic updates, it may not account for the knowledge graph’s long-term dependencies and complex relationships.

Utilizing convolutional operators for link prediction in knowledge graph embedding (KGE) presents a compelling solution to address the limitations of shallow models and fully connected deep architectures. These operators offer several key advantages, including parameter efficiency, scalability, robustness to overfitting, and flexibility. The parameter-efficient nature of convolutional operators allows for increased model expressiveness without the need to scale up the embedding size, which is particularly beneficial for large knowledge graphs where the number of embedding parameters is proportional to the number of entities and relations [26]. Additionally, convolutional operators’ highly optimized GPU implementations enable efficient computation, ensuring faster training and inference processes that can handle larger knowledge graphs without significantly increasing computational costs. Moreover, the established methodologies for controlling overfitting in multi-layer convolutional networks [27,28] can be applied to KGE link prediction tasks, effectively mitigating the overfitting issues commonly encountered in fully connected deep architectures. Lastly, composing convolutional operators into deep networks provides flexibility in designing more sophisticated models capable of capturing complex patterns and relationships within knowledge graphs. In conclusion, adopting convolutional operators for link prediction tasks in KGE offers a promising approach that addresses existing models’ challenges.

Existing convolutional algorithms primarily focus on mining triplet information, utilizing convolutional neural networks (CNNs) to take the embeddings of head entities and relations as input and predict tail entities. However, as demonstrated in ConvE [29], this approach cannot explore complex graph structure information between entities within triplets. The learning and parameter update processes treat each input triplet relatively independently, neglecting the entities’ connections.

To address this limitation, we propose incorporating graph structure information into the convolution by adding structural information about entities and their neighboring entities to improve model performance. Specifically, we construct edges based on co-occurrence relationships between entities in triplets or use a global graph structure to obtain the neighboring entity information for each entity. In addition to the original triplets, we introduce a new graph structure task incorporating the head entity’s neighboring information as new input during training, using a CNN to predict the corresponding tail entities. We utilize the average embedding of neighboring nodes for convolution. Furthermore, we provide the option to include edge information in the input of the convolutional model, allowing users to adjust the model’s structure and parameters based on their data.

The contributions of our work are as follows:

- We innovatively introduce convolutional operators to knowledge graph embedding (KGE) link prediction. This advancement bridges the gap between the shortcomings of shallow and densely connected architectures, harnessing the benefits of convolutional operators, such as parameter efficiency, superior scalability, robustness against overfitting, and the flexibility to craft intricate models deciphering complex relationships in knowledge graphs.
- We propose assimilating graph structure information into the convolutional framework. By leveraging edges constructed from co-occurrence patterns or a broader graph structure, our model incorporates the rich context of neighboring entity information. The introduction of a new graph structure task and the provision to integrate edge information in the convolutional input further bolster the model’s predictive prowess.

## 2. Related Work

This section reviews recent advancements in knowledge graph embeddings, particularly emphasizing the application of convolutional neural networks (CNNs), including Conv2D. Knowledge graph embeddings aim to represent entities and relations in a knowledge graph as continuous low-dimensional vectors. Numerous methods have been proposed, from traditional embedding techniques to graph-based neural networks.

### 2.1. Graph-Based Neural Networks for Knowledge Graph Embeddings

Traditional embedding approaches: Techniques such as TransE, DistMult, and ComplEx have laid the foundation in this domain. However, the focus has shifted towards capturing complex dependencies and patterns in the graph structure more effectively. Graph Convolutional Networks (GCNs) have gained popularity in the knowledge graph embedding domain. For instance, Liu et al. [30] integrated GCNs with attention mechanisms, while Zhang et al. [31] introduced a hierarchical structure into GCNs for capturing multi-scale features. Recent works, such as those by Li et al. [32,33], have expanded on these concepts, highlighting the versatility of graph-based neural networks in knowledge graph embeddings.

### 2.2. Applications of Convolutional Neural Networks in Various Domains

The application of CNNs, especially Conv2D, in knowledge graph embeddings has garnered interest. Wang et al. [34] and Liu et al. [35] have demonstrated the potential of CNNs in this domain.

Zhang et al. [36] introduced a hybrid convolutional spatial–temporal recurrent network for traffic flow prediction. While adept at capturing spatial and temporal characteristics of traffic data using a combination of convolutional and recurrent neural networks, their method is primarily tailored to traffic data. It may not directly apply to the intricacies of KGs. Y. et al. [37] focused on modeling relation paths for knowledge graph completion. Though innovative in understanding and predicting entities and relationships in KGs, their approach might struggle with multifaceted relationships and scalability. Lu et al. [38] delved into extracting and fusing multi-scale features of images and text for visual question answering. While their method enhances performance by integrating multiple feature extraction techniques, its application is primarily in visual data and might not be directly translatable to KGs. Guo et al. [39] proposed a path extension similarity link prediction method based on matrix algebra. Their approach, considering the directionality of networks, offers a fresh perspective but might encounter computational challenges when applied to large KGs. Di Wu et al. [40] introduced a graph-incorporated latent factor analysis model tailored for high-dimensional sparse data. By merging graph theory with factor analysis, their method captures data structures effectively. However, its application might be limited by the inherent complexities of integrating graph structures with latent factors. Di Wu et al. [41] presented a double-space and double-norm ensembled latent factor model for precise web service QoS prediction. Considering multiple service characteristics, their innovative approach might face challenges when applied outside web services.

In summary, while traditional embedding methods have laid the groundwork, recent research has delved deeper into the potential of graph-based neural networks and CNNs, especially Conv2D, in knowledge graph embeddings. This work aims to contribute to this growing body of research by proposing a novel Conv2D-based method for knowledge graph embeddings, considering the potential benefits of capturing local patterns, translation invariance, and hierarchical learning. Unlike the aforementioned methodologies, our approach amalgamates convolutional operators with pertinent graph structural information, offering a more comprehensive solution for link prediction in KGs. Our method captures intricate relationships between entities and provides flexibility in model architecture and parameter adjustments, outperforming existing benchmarks on multiple datasets.

## 3. Methodology

Most existing link prediction methods only use triplet-based knowledge, and they usually ignore the neighborhood subgraph knowledge of entities, which implies richer alignment information for aligning entities.

### 3.1. Problem Statement

A knowledge graph, denoted by G=(s,r,o)⊆E×R×E, can be defined as a set of triples (facts), with each triple consisting of a relationship r∈R and two entities s,o∈E. The entities are referred to as the subject and object of the triple, respectively. Each triple (s,r,o) represents a relationship of type *r* between entities *s* and *o*.

The link prediction problem can be framed as a pointwise learning-to-rank problem, where the objective is to learn a scoring function ψ:E×R×E↦R. Given an input triple x=(s,r,o), its corresponding score ψ(x)∈R is proportional to the likelihood that the fact encoded by *x* is true.

Neural link prediction models are multi-layer neural networks comprising encoding and scoring components. Given an input triple (s,r,o), the encoding component maps entities s,o∈E to their distributed embedding representations $es,eo\in R^{k}$. We present scoring functions $\psi r\left(es,eo\right)$ from the neural link predictors found, along with their relation-dependent parameters and space complexity. Here, ne and nr denote the number of entities and relation types, respectively, such that ne=|E| and $n_{r}=\left|R\right|$. In the scoring component, the two entity embeddings es and eo are evaluated by a function ψr. The score of a triple (s,r,o) is defined as $\psi \left(s,r,o\right)=\psi r\left(e_{s},e_{o}\right)\in R$.

### 3.2. Convolutional 2D Knowledge Graph Embeddings

**Figure 1:**
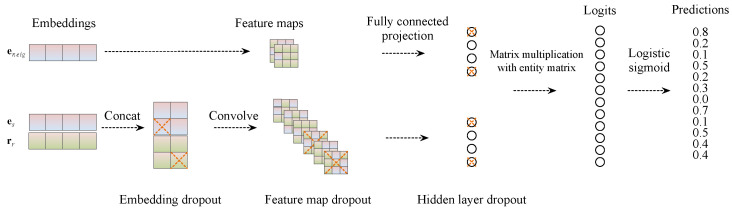
Here depicted is our model architecture. The entity and relation embeddings are initially reshaped and concatenated while considering the neighboring nodes concurrently. This resultant matrix subsequently serves as the input for a convolutional layer. The resulting feature map tensor is then vectorized and projected into a k-dimensional space, where it is matched with all potential object embeddings.

As shown in Figure 1, this work proposes a neural link prediction model in which convolutional and fully connected layers model interactions between input entities and relationships. Specifically, the neighbor entity information of each entity is obtained based on the entity co-occurrence relationship in the triple information or the global graph structure. Based on the original triplet, a new graph structure task is introduced; that is, the neighbor information of the head entity is used as a new input during training, and the corresponding tail entity is predicted using a convolutional neural network. This provides two schemes: direct convolution with neighbor nodes and convolution with the average embedding of neighbor nodes.

#### 3.2.1. Motivation for Incorporating Neighboring Information

Entities within a knowledge graph are not isolated nodes; they inherently form contexts with their neighbors. The motivation for incorporating this neighboring information arises from several core insights:

1. Richer semantic capturing: Each entity’s relationship with its neighbors provides valuable semantic information that is otherwise overlooked if only direct embeddings are used. By tapping into this, we ensure that subtler, context-specific nuances in relationships are captured.
2. Enhanced predictive power: Knowledge graphs often have complex and interwoven relationships. Considering the surrounding context (i.e., neighboring entities), our model gains more predictive power, especially in densely interconnected graph regions where simple entity–relation–entity predictions might be ambiguous.
3. Robustness to sparse data: In scenarios where certain entities have limited direct relationships, leveraging neighboring information can supplement the lack of direct data, making predictions more robust and informed.
4. Model generalization: Incorporating neighboring information can lead to better generalization. By understanding the broader context in which an entity exists, the model is less likely to overfit specific triples and can generalize better to unseen or rare triples.
5. Handling dynamic knowledge graphs: Entities may form new relationships as knowledge graphs evolve. A model cognizant of neighboring contexts can adapt more swiftly to such changes, ensuring that predictions remain relevant even as the graph’s topology evolves.

While direct embeddings provide a snapshot of individual entities and their relations, neighboring information offers a panoramic view, placing entities within a broader, interconnected context. This holistic perspective is pivotal in crafting a more comprehensive and nuanced representation, indispensable for robust link prediction in knowledge graphs.

#### 3.2.2. Extraction and Processing of Neighbor Information

Neighbor entity information for each entity is extracted based on entity co-occurrence relationships within triples or from the overall graph structure. For the said neighboring information, we contemplate two strategies:

- Direct convolution with neighbor nodes.
- Convolution with the average embedding of neighbor nodes, wherein we first calculate embeddings for each neighbor and then compute their average. This is designed considering potential weight differences amongst neighbors.

The main feature of our model is that a convolution over 2D-shaped embeddings determines the score. The scoring function is formally defined as follows:

(1) $$\psi_{r}\left(e_{s},e_{o}\right)=f\left(\mathrm{vec}\left(f(\left[\bar{e_{s}};\bar{r_{r}}\right]*\omega \right)\right)W)e_{o},$$

(2) $$\psi_{r}\left(e_{sneig},e_{o}\right)=f\left(\mathrm{vec}\left(f(\left[\bar{e_{sneig}};\bar{r_{r}}\right]*\omega \right)\right)W)e_{o},$$

(3) $$\psi_{r}=\omega_{1}\cdot \psi_{r}\left(e_{s},e_{o}\right)+\omega_{2}\cdot \psi_{r}\left(e_{sneig},e_{o}\right)$$

where f(·) refers to the convolution operation, $e_{sneig}$ represents the neighbor node of node *s*, $r_{r}\in R^{k}$ is a relation parameter dependent on *r*. The symbols $\bar{e_{s}}$ and $\bar{r_{r}}$ represent 2D reshaped versions of $e_{s}$ and $r_{r}$, respectively. If $e_{s},r_{r}\in R^{k}$, then $\bar{e_{s}},\bar{r_{r}}\in R^{kw\times k_{h}}$, where $k=k_{w}k_{h}$.

#### 3.2.3. Detailed Feed-Forward Process

During the feed-forward pass, the model carries out a row–vector lookup operation on two embedding matrices: one for entities, denoted by $E^{|E|\times k}$, and the other for relations, denoted by $R^{\left|R\right|\times k^{\prime }}$. Here, *k* and $k^{\prime }$ represent the entity and relation embedding dimensions, while |E| and |R| signify the numbers of entities and relations, respectively. The model concatenates $\bar{e_{s}}$ and $\bar{r_{r}}$ and uses the result as input for a 2D convolutional layer with filters ω. This layer produces a feature map tensor $T\in R^{c\times m\times n}$, where *c* is the number of 2D feature maps with dimensions *m* and *n*. Tensor T is then reshaped into a vector $\mathrm{vec}\left(T\right)\in R^{cmn}$, which is subsequently projected into a *k*-dimensional space using a linear transformation parameterized by matrix $W\in R^{cmn\times k}$. Object embedding $e_{o}$ is then matched using an inner product. The parameters of the convolutional filters and matrix W are independent of the parameters for entities *s* and *o*, as well as relationship *r*.

#### 3.2.4. Rationale behind 2D Convolution

In the realm of knowledge graphs, where the latent relationships are deeply embedded within high-dimensional spaces, utilizing 2D convolutions gives multiple advantages:

1. Pattern recognition: Traditional embeddings, while effective, may fail to capture intricate patterns when considering higher dimensions. Two-dimensional convolutions excel in identifying localized patterns within embeddings, which better captures nuanced relationships between entities in the context of knowledge graphs.
2. Spatial hierarchies: Two-dimensional convolutional layers can identify hierarchical structures within the embedding space. This is particularly important in knowledge graphs, where relationships can have hierarchical or layered nuances. For instance, “being a part of” versus “being affiliated with” might manifest differently in the embedding space, and 2D convolutions can tease these differences.
3. Parameter efficiency: By reshaping embeddings into 2D structures and applying convolutions, the model can capture spatial relationships with fewer parameters than fully connected layers. This can lead to faster training and less overfitting.
4. Translational invariance: One of the hallmark features of convolutional layers is their ability to detect features irrespective of their position in the input. In the context of our embeddings, this ensures that important relational cues are captured irrespective of their positioning within the high-dimensional space.
5. Adaptive feature learning: Two-dimensional convolutions automatically learn features from the data rather than relying on handcrafted features. This adaptability is essential in knowledge graphs, where the diversity of relationships and entities can be vast and unpredictable.

While traditional embeddings capture a static representation of entities and relationships, 2D convolutions breathe life into these embeddings by capturing dynamic and spatially relevant patterns. This added layer of analysis is instrumental in accurately understanding and predicting the rich tapestry of relationships present in knowledge graphs.

### 3.3. Loss Function

To train the model parameters, we apply the logistic sigmoid function, σ(·), to the scores, such that $p=\sigma \left(\psi_{r}\left(es,eo\right)\right)$, and minimize the following binary cross-entropy loss:

(4) $$L\left(p,t\right)=-\frac{1}{N}\sum_{i}\left(t_{i}\cdot \log\left(p_{i}\right)+\left(1-t_{i}\right)\cdot \log\left(1-p_{i}\right)\right),$$

where *t* is the label vector with dimension $R^{1\times 1}$ for 1−1 scoring or $R^{1\times N}$ for 1−N scoring (refer to the next section for 1−N scoring). The elements of vector *t* are ones for existing relationships and zeros otherwise.

## 4. Experiments

### 4.1. Knowledge Graph Datasets

We assess our proposed model using a variety of link prediction datasets from the literature.

**WN18** [42] is a subset of WordNet comprising 18 relations and 40,943 entities. Most 151,442 triples are based on hyponym and hypernym relations, giving WN18 a primarily hierarchical structure. However, due to its composition, WN18 contains many inverse relations, which allows some models to achieve high accuracy simply by memorizing these inverse relations rather than truly learning meaningful representations. To address this, **WN18RR** was introduced, removing these easy-to-memorize inverse relations and offering a more challenging model benchmark.

**FB15k** [42] is a subset of Freebase that contains 14,951 entities and 1345 distinct relations. Much of this knowledge graph is dedicated to facts about movies, actors, awards, sports, and sports teams. While it serves as a common evaluation benchmark, researchers identified data leakage issues among its training, validation, and test sets that might inflate the performance of models. To remedy this, **FB15k-237** excluding triples that could lead to leakage was proposed, providing a fairer and more challenging evaluation standard.

**YAGO3-10** [43] is a YAGO3 subset consisting of entities with at least 10 relations each. It includes 123,182 entities and 37 relations, with most triples concerning descriptive attributes of people, such as citizenship, gender, and profession.

### 4.2. Experimental Setup

We employ rectified linear units (ReLUs) as the non-linearity *f* for accelerated training and use batch normalization after each layer to stabilize, regularize, and improve convergence speed. Our model is regularized by incorporating drop out at various stages, specifically on the embeddings, the feature maps following the convolution operation, and the hidden units after the fully connected layer. We utilize the Adam optimizer and implement label smoothing to mitigate overfitting caused by output non-linearity saturation at the labels.

### 4.3. Results and Analysis

Table 1 presents the link prediction results for the WN18 and FB15k datasets. Multiple benchmark algorithms, including TransE, DistMult, CompEx, Gaifman, ANALOGY, R-GCN, and ConvE, are evaluated alongside our proposed methodology. Key metrics used for this assessment are Mean Rank (MR), Mean Reciprocal Rank (MRR), and Hits@n for n = 10, 3, and 1.

Our methodology outperforms all other techniques on the WN18 dataset regarding MRR with a score of 0.954. The closest competitor is ConvE, with a score of 0.943. Our method also achieves the lowest MR, 293, suggesting that our predicted entities tend to rank closer to the top. For the Hits@10 metric, our approach, with a score of 0.962, is narrowly outperformed by R-GCN, which has a score of 0.964. However, our method surpasses all other methods in Hits@3 and Hits@1 metrics, demonstrating its effectiveness in ranking the correct entities very high in the predictions.

On the FB15k dataset, our method shines again. It achieves the lowest MR at 47, indicating that our predictions rank the correct entities near the top significantly better than the competitors. For MRR, our method scores 0.717, coming in second to ANALOGY, which has the highest MRR, 0.725. Nevertheless, our method outstrips all other methods in Hits@10, Hits@3, and Hits@1, with scores of 0.884, 0.788, and 0.711, respectively. It is worth noting the significant improvement our approach exhibits in the Hits@1 metric, registering a score of 0.711, compared with Gaifman, the next best, which scores 0.692.

The results underscore the superiority of our approach, especially when considering the breadth and depth of the relationships it captures. Including convolutional operators integrated with graph structural information provides a comprehensive understanding of the entities and their relationships, thus enhancing prediction accuracy. The consistent performance across different datasets, such as WN18 and FB15k, validates our methodology’s robustness and general applicability.

Table 2 delineates the results for link prediction on the WN18RR and FB15k-237 datasets. Multiple benchmark models, including TransE, DistMult, ComplEx, R-GCN, and ConvE, are juxtaposed against our proposed approach. The metrics employed for evaluation are Mean Rank (MR), Mean Reciprocal Rank (MRR), and Hits@n (where n = 10, 3, and 1).

Our proposed methodology sets a new benchmark on the WN18RR dataset. Regarding MRR, our approach achieves a score of 0.47, overtaking the closest competitor, ComplEx, which has a score of 0.44. Our method realizes the lowest MR, standing at 3245, which implies that our predictions rank the correct entities closer to the top, comparatively superior to other methods. While ConvE leads the pack in the Hits@10 metric with a score of 0.52, our method matches this performance, also scoring 0.51. Importantly, our approach outstrips other techniques in the Hits@3 and Hits@1 metrics, underscoring its robustness in pinpointing the correct entities.

Our methodology showcases dominance on the FB15k-237 dataset as well. The method’s MR is exceptional, landing at 189, making it the front-runner in positioning the correct entities at higher ranks. Regarding MRR, our method clinches the top position with a score of 0.427, considerably better than the runner-up ConvE, which records 0.325. Our approach’s supremacy is further evident in Hits@n metrics, as it consistently surpasses other models across Hits@10, Hits@3, and Hits@1. The following are general observations.

Table 3 portrays the link prediction outcomes on the YAGO3-10 dataset. Our proposed approach sets a precedent in the YAGO3-10 dataset as well. With respect to the MR metric, our method establishes a new benchmark, with a score of 1396. This suggests that our predictions place the correct entities considerably higher in rank, showcasing superior performance compared with the other models. The closest competing model is ConvE, which records an MR value of 1676. In the MRR evaluation, our methodology emerges as the leader, with a score of 0.47, outstripping ConvE’s second-best score of 0.44. The supremacy of our technique is further emphasized in the Hits@n metrics. Our method consistently leads, recording scores of 0.65, 0.54, and 0.43 for Hits@10, Hits@3, and Hits@1, respectively. In each of these metrics, our methodology eclipses the results of the benchmark models, solidifying its robustness and inaccurate predictions.

The YAGO3-10 results further corroborate the effectiveness of our methodology, showcasing its capability to position correct entities higher in the ranks and achieve higher accuracy in predictions. Our approach’s lead across all metrics accentuates the value of amalgamating convolutional operators with graph structural details, especially when applied to diverse datasets.

The evident success of our method on three datasets reaffirms the potency of integrating convolutional operators with graph structural details. The consistent, leading performance in multiple metrics demonstrates the comprehensive capability of the approach, from ranking correct entities closer to the top to ensuring higher accuracy in predictions. The contrast between our results and benchmark methods accentuates our approach’s knowledge graph embedding and link prediction improvement.

## 5. Ablation Study

To validate the effectiveness of our proposed method, we conducted a series of ablation experiments to systematically examine the influence of various parameters on the model’s performance. Through these experiments, we evaluated the impact of different neighbor node aggregation, mean pooling, and neighbor node convolution on the model’s performance.

Drawing from the analytical insights given in Table 4, our focus veers towards the pivotal role of neighbor aggregation during the convolutional process. This, in essence, pertains to the systematic incorporation of proximate nodal information.

Our empirical findings elucidate a notable increase in the model’s efficacy when these neighboring contextual data are integrated relative to scenarios that eschew such inclusions. This conspicuously underscores the instrumental role of immediate nodal surroundings in crafting a richer, more semantically robust representation.

The zenith of our model’s performance was attained when conditioned on the amalgamation of both the direct entity representation and the structural nuances of its contiguous entities. This furnishes empirical credence to the hypothesis that concurrent inclusion of local (direct entity) and global (neighboring entities) contextual cues can beget superior model fidelity.

Interestingly, a narrowed focus solely on neighboring nodes—devoid of the principal entity’s context—still resulted in commendable outcomes, positioning them as the second most efficacious. This underlines the cardinal importance of neighborhood-derived semantics.

However, our investigative journey was not devoid of anomalies. The model’s performance trajectory evidenced a decline upon introducing relation nodes. One postulation attributes this decrement to the inadvertent infusion of noise or, perhaps, tangential data from these relation nodes. Such superfluous information could potentially occlude or dilute the primary semantics derived from the focal entity and its neighbors, engendering a sub-optimal or skewed contextual representation. This observation accentuates the delicate equilibrium that researchers must strike: seamlessly integrating germane contextual cues while circumventing the pitfalls of information redundancy from relation nodes.

### The Effect of Parameters

**Figure 2:**
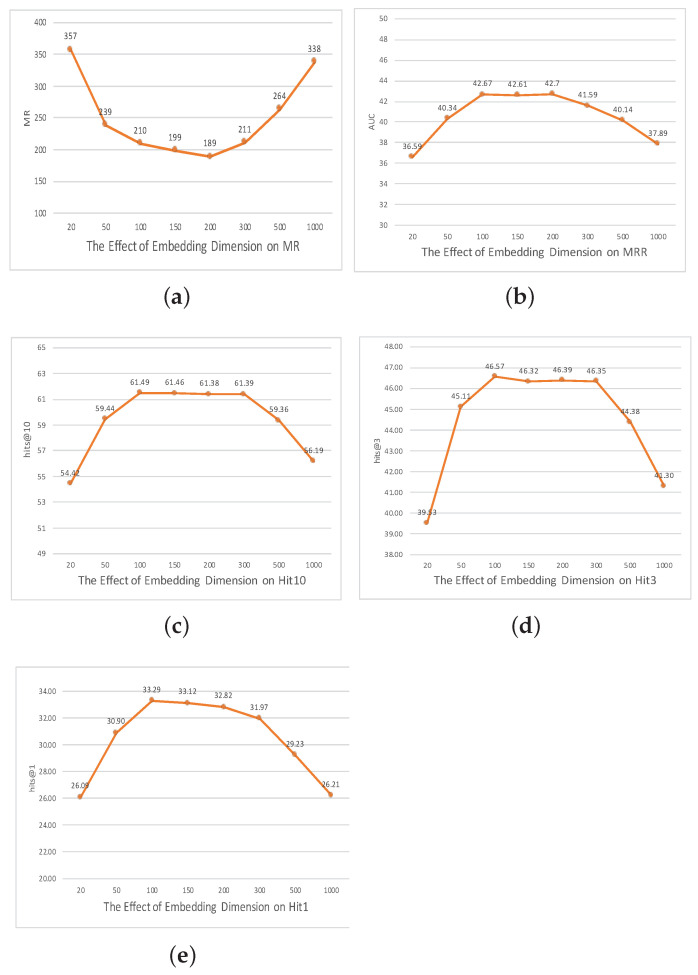
The effect of embedding dimensions on the FB15K237 dataset.

**Figure 3:**
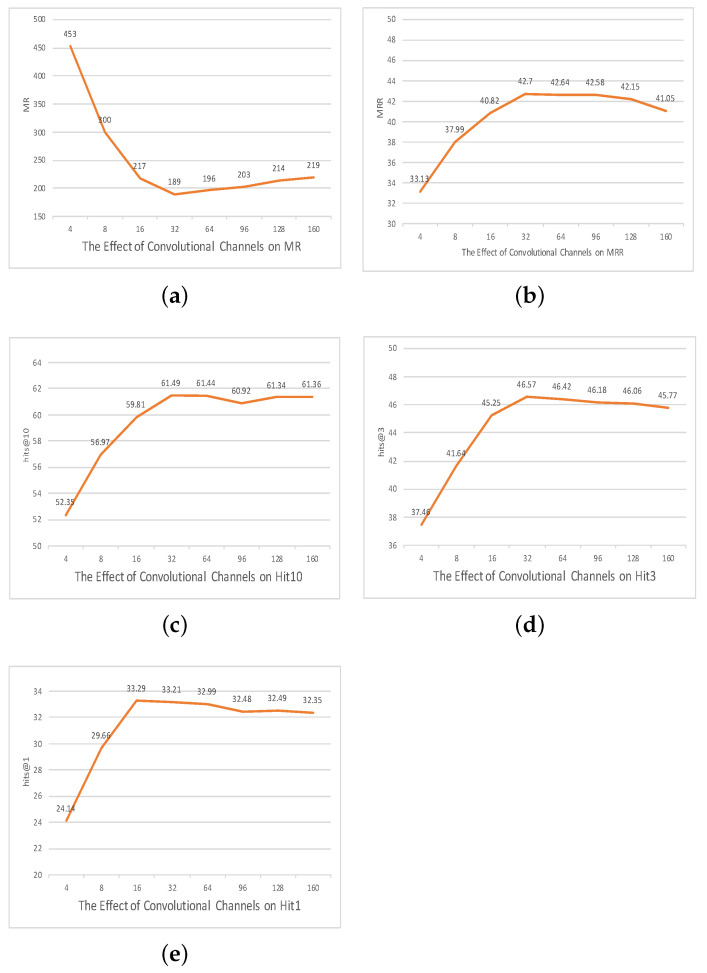
The effect of convolutional channels on the FB15K237 dataset.

**Figure 4:**
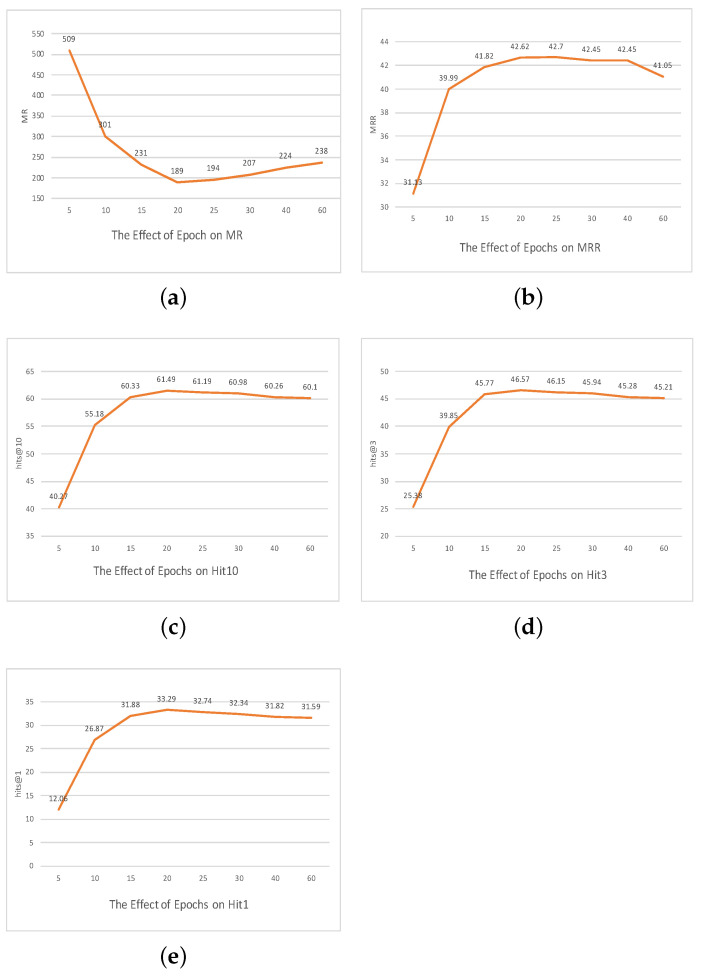
The effect of epochs on the FB15K237 dataset.

To rigorously elucidate the interplay between different hyperparameters and the resultant efficacy of our model, as shown in Figure 2, Figure 3 and Figure 4, we embarked on a comprehensive ablation study. This exploration centered on three pivotal axes: embedding dimensionality, the capacity of the convolutional channels, and the span of training epochs.

**Embedding dimensionality and its implications:** The dimensionality of embeddings is inherently tied to their expressiveness, acting as a conduit that encodes intricate relationships and complex patterns within the knowledge graph. Intuitively, richer (higher-dimensional) embeddings potentially offer a more nuanced representation. However, this also raises the specter of model overfitting and computational inefficiency.

To empirically quantify the trade-offs, we undertook experiments across a spectrum of embedding dimensions: 20, 50, and 100, culminating at 1000. Our findings revealed an optimal point at the dimensionality of 200. At this juncture, the model’s performance in the MR task was unrivaled. This observation suggests that the dimensionality 200 harmonizes the dichotomy of embedding richness and model generalization, ensuring that while the model captures the requisite granularity, it remains immune to overfitting.

**Influence of convolutional channel capacity:** Convolutional channels act as the conduits for feature extraction in neural architectures. Their capacity, thus, inherently affects the granularity and the diversity of features that the model discerns from the input. We conducted expansive experiments over various channel dimensions from 4 to 160 to discern the inflection point where the model maximizes its predictive acumen.

Our empirical results pinpointed 32 as the optimal number of channels. The elucidation of this observation can be anchored in the following intricacies.

*Feature extraction depth versus overfitting risk:* While increasing the number of channels intuitively amplifies the model’s ability to unearth a diverse set of patterns, a limit exists beyond which these gains are offset. Specifically, the model might veer into overfitting after a certain saturation point, becoming unduly tailored to training nuances and losing its generalization capabilities. Per our experiments, the threshold of 32 channels appears to be the juncture where the model extracts a rich set of features without succumbing to the overfitting pitfall.

*Marginal gains and computational trade-offs:* Analogous to our insights from the embedding dimension analysis, the convolutional channel capacity also exhibits a diminishing returns phenomenon. Each incremental increase in channel dimensions yields progressively marginal enhancements in performance. Beyond the 32-channel mark, our findings indicated that any minuscule gains in performance were overshadowed by the computational overhead and increased risk of model overcomplexity.

Given the aforementioned observations, our model seems to flourish in an environment with 32 convolutional channels, striking an optimal balance between robust feature extraction and computational efficiency.

**The impact of training epochs:** The longevity of a model’s training cycle—denoted by the number of epochs—plays an instrumental role in shaping its learning trajectory and eventual predictive prowess. To demystify the relationship between the training duration and the resultant model accuracy, we instituted experiments across various epoch durations, spanning from a minimalistic 5 epochs to an extended 60 epochs.

Our empirical diagnostics flagged 20 epochs as the sweet spot, and a deeper dive into the dynamics of training epochs elucidates the following observation.

*The convergence dilemma:* The trajectory of model training is fundamentally underpinned by its convergence behavior. Training epochs too scanty in number might truncate the model’s learning process prematurely, ensnaring it in the realms of underfitting. Conversely, an overextended training duration risks overfitting, as the model might transition from discerning patterns to rote memorization of training intricacies. Our investigative foray revealed that at the 20-epoch mark, the model consummates a learning journey that adeptly bridges the chasm between underfitting and overfitting.

*Performance vs. computational expediency:* Beyond the pure predictive acumen, the operational efficiency of a model, encapsulated by its training duration, holds paramount importance. Each epoch incrementally escalates the computational and temporal investments, but the corresponding upticks in performance often follow a law of diminishing returns. Our experiments illuminated that the 20-epoch configuration championed a harmonious synergy between computational expediency and model accuracy.

In summation, within our experimental landscape, the training dynamics orchestrated over 20 epochs manifested as an optimal regimen, balancing the intricacies of model learning, generalization, and computational pragmatism.

## 6. Conclusions

We have introduced a link prediction model that leverages convolutional techniques applied to embeddings, incorporating multiple layers of non-linear features to model knowledge graphs effectively. Our approach stands out for several reasons: it takes advantage of neighbor node information, maintains expressiveness through multiple layers of non-linear features, demonstrates robustness against overfitting thanks to batch normalization and dropout, and consistently achieves superior results across various datasets. Our analysis indicates that the improved performance of our model when compared to a common link predictor like DistMult, can be partially attributed to its capacity to effectively model nodes with high (recursive) degrees.

While our model has already exhibited promising results, it remains relatively shallow compared with the convolutional architectures typically encountered in computer vision applications. Future research endeavors may focus on deepening the convolutional models we employ. Furthermore, there is room for exploring the implications of convolution within our context. Additionally, we may investigate methods for enforcing large-scale structural patterns within the embedding space to facilitate increased interactions among embeddings.

## Figures and Tables

**Table 1:** Link prediction results for WN18 and FB15k.

	WN18					FB15k				
				**Hits**					**Hits**	
	**MR**	**MRR**	**@10**	**@3**	**@1**	**MR**	**MRR**	**@10**	**@3**	**@1**
TransE [42]	-	0.495	0.943	0.888	0.113	-	0.463	0.749	0.578	0.297
DistMult [22]	902	0.822	0.936	0.914	0.728	97	0.654	0.824	0.733	0.546
CompEx [23]	-	0.941	0.947	0.936	0.936	-	0.692	0.840	0.759	0.599
Gaifman [44]	352	-	0.939	-	0.761	75	-	0.842	-	0.692
ANALOGY [45]	-	0.942	0.947	0.944	0.939	-	**0.725**	0.854	0.785	0.646
R-GCN [46]	-	0.814	**0.964**	0.929	0.697	-	0.696	0.842	0.760	0.601
ConvE [29]	374	0.943	0.956	0.946	0.935	51	0.657	0.831	0.723	0.558
Ours	**293**	**0.954**	0.962	**0.951**	**0.942**	**47**	0.717	**0.884**	**0.788**	**0.711**

**Table 2:** Link prediction results for WN18RR and FB15k-237.

	WN18RR					FB15k-237				
				**Hits**					**Hits**	
	**MR**	**MRR**	**@10**	**@3**	**@1**	**MR**	**MRR**	**@10**	**@3**	**@1**
TransE [42]	-	0.23	0.52	0.36	0.06	-	0.310	0.495	0.345	0.218
DistMult [22]	5110	0.43	0.49	0.44	0.39	254	0.241	0.419	0.263	0.155
ComplEx [23]	5261	0.44	0.51	0.46	0.41	339	0.247	0.428	0.275	0.158
R-GCN [46]	-	-	-	-	-	-	0.248	0.417	0.258	0.153
ConvE [29]	4187	0.43	**0.52**	0.44	0.40	244	0.325	0.501	0.356	0.237
Ours	**3245**	**0.47**	0.51	**0.47**	**0.44**	**189**	**0.427**	**0.615**	**0.466**	**0.333**

**Table 3:** Link prediction results for YAGO3-10.

	YAGO3-10				
			**Hits**		
	**MR**	**MRR**	**@10**	**@3**	**@1**
DistMult [22]	5926	0.34	0.54	0.38	0.24
ComplEx [23]	6351	0.36	0.55	0.40	0.26
ConvE [29]	1676	0.44	0.62	0.49	0.35
Ours	**1396**	**0.47**	**0.65**	**0.54**	**0.43**

**Table 4:** Ablation study.

	Neighbor Aggregation	Relation	Neighbor Convolution	Mean Pooling	MR	MRR	Hits10	Hits3	Hits1
raw					233.73	0.4041	0.6003	0.4468	0.3041
raw+agg+conv	✓		✓		201.61	0.4234	0.6083	**0.4657**	0.3285
raw+agg+conv+relation	✓	✓	✓		256.05	0.4090	0.5942	0.4506	0.3156
raw+agg+pooling+relation	✓	✓		✓	216.94	0.4073	0.5980	0.4452	0.3134
raw+agg+pooling	✓			✓	**189.25**	**0.4270**	**0.6149**	**0.4657**	**0.3329**

## Data Availability

Not applicable.
